# Lack of association between mutations of gene-encoding mitochondrial D310 (displacement loop) mononucleotide repeat and oxidative stress in chronic dialysis patients in Taiwan

**DOI:** 10.1186/1477-5751-8-10

**Published:** 2009-11-05

**Authors:** Jin-Bor Chen, Tsu-Kung Lin, Shang-Chih Liao, Wen-Chin Lee, Lung-Chih Lee, Chia-Wei Liou, Pei-Wen Wang, Mao-Meng Tiao

**Affiliations:** 1 Nephrology Division, Chang Gung Memorial Hospital-Kaohsiung Medical Center, Chang Gung University College of Medicine, Taiwan; 2 Department of Neurology, Chang Gung Memorial Hospital-Kaohsiung Medical Center, Chang Gung University College of Medicine, Taiwan; 3 Endocrine Division, Chang Gung Memorial Hospital-Kaohsiung Medical Center, Chang Gung University College of Medicine, Taiwan; 4 Department of Pediatrics, Chang Gung Memorial Hospital-Kaohsiung Medical Center, Chang Gung University College of Medicine, Taiwan

## Abstract

**Background:**

Mitochondria (mt) are highly susceptible to reactive oxygen species (ROS). In this study, we investigated the association between a region within the displacement loop (D-loop) in mtDNA that is highly susceptible to ROS and oxidative stress markers in chronic dialysis patients. We enrolled 184 chronic dialysis patients and 213 age-matched healthy subjects for comparison. Blood levels of oxidative stress markers, such as thiobarbituric acid reactive substances (TBARS) and free thiol, and the mtDNA copy number were determined. A mononucleotide repeat sequence (CCCC...CCCTCCCCCC) between nucleotides 303 and 316-318 (D310) was identified in mtDNA.

**Results:**

Depending on alterations in the D310 mononucleotide repeat, subjects were categorized into 4 subgroups: 7-C, 8-C, 9 or 10-C, and T-to-C transition. Oxidative stress was higher in chronic dialysis patients, evidenced by higher levels of TBARS and mtDNA copy number, and a lower level of free thiol. The distribution of 7-C, 8-C, and 9-10C in dialysis and control subjects was as follows: 7-C (38% *vs. *31.5%), 8-C (35.3% *vs. *43.2%), and 9-10C (24.5% *vs. *22.1%). Although there were significant differences in levels of TBARS, free thiol, and the mtDNA copy number in the D310 repeat subgroups (except T-to-C transition) between dialysis patients and control subjects, post hoc analyses within the same study cohort revealed no significant differences.

**Conclusion:**

Although oxidative stress was elevated in chronic dialysis patients and resulted in a compensatory increase in the mtDNA copy number, homopolymeric C repeats in the mtDNA region (D310), susceptible to ROS, were not associated with oxidative stress markers in these patients.

## Background

Chronic kidney disease (CKD) is characterized by a state of increased oxidative stress [1]. This has been evidenced by changes in oxidative stress biomarkers in the blood and in tissue DNA. In our previous study, we demonstrated that, in continuous ambulatory peritoneal dialysis (CAPD) patients, the level of oxidative stress biomarkers in the blood was elevated, with an accompanying increase in the copy number of mitochondrial (mt) DNA [2]. mtDNA is a 16.6-kb double-stranded closed-circular DNA molecule. In humans, the mutation rate of mtDNA is at least 10 times higher than that of nuclear DNA. Most mutations in mtDNA accumulate in the displacement loop (D-loop) region. The D-loop functions as a promoter for both heavy and light strands of mtDNA and does not encode any functional proteins. A mononucleotide repeat sequence (CCCC...CCCTCCCCCC) between nucleotides 303 and 316-318 of mtDNA has been reported to be a hotspot of deletion or insertion mutation in primary tumors [3-5]. D310 has been indicated to this region, a term previously assigned by other investigators [6]. The first stretch of cytosine bases is highly polymorphic, ranging from 7-C and 9-C; 7-C is the most common sequence. The D310 repeat is a part of conserved sequence block II (CSBII), located 92 bp from the heavy-strand replication region, and mtDNA replication is initiated when CSBII forms a persistent RNA-DNA hybrid with CSBI and CSBIII regions [7].

Nucleotide sequence analysis revealed that only a homopolymeric sequence in CSBII, ranging from 6 to 12 residues in length, showed variability. *In vitro *transcriptional analyses have revealed that most common polymers could support accurate transcriptional initiation [8]. Because the D-loop region is a susceptible region for oxidative stress, we hypothesized that the D-loop mutation exists in dialysis patients with an increased oxidative stress status. In this study, we investigated that hypothesis via measurement of the D310 repeat in dialysis patients.

## Methods

As study subjects, we enrolled those patients who visited our outpatient department between December 2004 and July 2005. Dialysis patients were considered eligible if they had been receiving CAPD and hemodialysis (HD) for more than 3 months; showed no evidence of chronic or acute infections, inflammatory disorders, and malignancy; and were not using anti-inflammatory drugs for 3 months before enrollment. Of the 184 dialysis patients who were enrolled, there were 88 CAPD patients and 96 HD patients. Among the dialysis cohort, 22 patients were diabetics. The CAPD patients underwent dialysis with commercially available dialysate (Dianeal PD solution; Baxter, Singapore) containing 40 mmol/L lactate (pH 5.2). The HD patients underwent HD 3 times a week with the same type of HD membrane for more than 3 months. A modified cellulose membrane (Toray, Tokyo, Japan) or a synthetic membrane (polysulfone; Fresenius, Borkenberg, Germany) was used. The dialysate was bicarbonate-based and dextrose-free with 2.0 mEq/L potassium, 140 mEq/L sodium, and 3.0 mEq/L calcium. Dialysate and blood flow rates were 500 mL/min and 250-300 mL/min, respectively. We also selected 213 age-matched healthy subjects from the health center at our hospital. This control group had normal renal function defined by a creatinine clearance of >100 mL/min. Healthy subjects with systemic diseases, such as diabetes mellitus, renal disease, hepatic disease, cardiac disease, and hypertension were excluded.

Venous blood samples of all subjects were obtained after they had fasted for at least 8 h. Blood samples were processed immediately. Levels of serum albumin, glucose, total cholesterol, triglyceride, and electrolytes were measured with an autoanalyzer using commercial kits (Hitachi 7600.210; Hitachi Ltd., Tokyo, Japan). Biochemical data were also obtained. Blood collected in tubes containing ethylenediaminetetraacetic acid (EDTA) was centrifuged at 1,500 *g *for 10 min at 4°C, and mtDNA was extracted from peripheral leukocytes. The protocol was approved by the Committee on Human Research at Chang Gung Memorial Hospital-Kaohsiung Medical Center. Informed consent was obtained from all participants.

### Estimation of plasma free thiol content

Plasma-free thiol content was measured by directly reacting thiols with 5, 5-dithiobis 2-nitrobenzoic acid (DTNB), whereby 5-thio-2-nitrobenzoic acid (TNB) was formed [9]. Thiol content of the sample was calculated from the absorbance by using the extinction coefficient of TNB (A_412 _= 13,600 M^-1^·cm^-1^) [9].

### Estimation of plasma content of thiobarbituric acid reactive substances)

The plasma content of thiobarbituric acid reactive substances (TBARS) was assessed by the method described by Ohkawa et al. [10]. After centrifugation, plasma samples were stored at -80°C for further analysis. The results were expressed in terms of micromoles of TBARS per liter. A standard curve of TBARS was obtained by hydrolysis of 1,1,3,3-tetraethoxypropane.

### Determination of relative leukocyte mtDNA copy number

#### DNA purification

Blood was collected in EDTA (1 mg/ml)-containing tubes from the peripheral vein. Buffy coat was separated from blood samples by centrifugation for 10 min at 3,000 rpm. The supernatant was carefully discarded by pipetting. DNA was extracted using the Gentra^® ^DNA extraction kit (Qiagen, Germany).

### Determination of mtDNA content

For determination of mtDNA content relative to nuclear DNA, the forward primer5'-GGCTCTGTGAGGGATATAAAGACA-3' and reverse primer 5'-CAAACCAC CCGAGCAACTAATCT-3' [complementary to sequences of chromosome 1 (Chr1) genome loci on 1q24-25] were used to amplify a 97-bp product.

For analysis of mtDNA, we used *ND2 *gene sequences, the forward primer 5'-CACAGAAGCTGCCATCAAGTA-3' and reverse primer 5'-CCGGAGAG TATATTGTTGAAGAG-3', were amplified to obtain an 89-bp product. Polymerase chain reaction (PCR) was performed in a Roche Lightcycle^® ^480r (Roche Applied Sciences, Mannheim, Germany) apparatus using the LightCycler^® ^480 SYBR Green I Master Mix kit (Roche Applied Science). DNA (10 ng) was mixed with 10 μl LightCycler^® ^480 SYBR Green I Master Mix containing 5 μmol (final concentration 0.4 μM) of forward and reverse primers in a final volume of 20 μl. PCR reactions were conducted as follows: initially at 50°C for 2 min, 95°C for 1 min, 40 cycles for denaturation at 95°C for 15 s, annealing at 60°C for 20 s, and primer extension at 72°C for 15 s, and finally at 25°C. The threshold cycle number (Ct) values of the *Chr1 *gene and *ND2 *gene were determined for each individual quantitative PCR run. The equation ΔCt [Ct (ND2)-Ct (Chr1)] represents the relative abundance. The quantitative results were expressed as the copy number of the mtDNA/cell by 2 × 2^-ΔCt^. Each measurement was carried out at least 3 times and normalized in each experiment against a serial dilution series of a control DNA sample.

### Determination of D-loop sequence

The mtDNA control region segment (corresponding to region 15911-602 in the Cambridge Reference Sequence) was amplified using L15911 forward primer (5'-ACCAGTCTTGTAAACCGGAG-3') and H602 reverse primer (5'-GCTTTGAGGAGGTAAGCTAC-3'). The products were purified with the PCR Product Pre-Sequencing Kit (GE Healthcare Life Sciences, Sweden) and sequenced by using primer L15911 and primer L29 (5'-CTCACGGGAGCTCnTCCATGC-3') on an ABI 377XL DNA sequencer (Applied Biosystems). However, because of the frequent occurrence of poly-C at nucleotide pairs 16189 and 310 within the D-loop region, the sequencing procedure ceased every time the samples harbored these variants. Reverse sequencing was then used with 2 additional primers, namely, H81 (5'-CAGCGTCTCGCAATGCTATC-3') and H602 (5'-GCTTTGAGGAGGTAAGCTAC-3'). DNA sequences were analyzed by DNASTAR and BioEdit sequencing alignment editor software (Copyright 1997-2007 Tom Hall).

### Statistical analysis

Statistical analysis was performed using the SPSS^® ^version 12.0 software (SPSS Inc., Chicago, IL, USA). Before analysis, all pre-exam data were fitted into a normal distribution. A two-tailed one-way analysis of variance (ANOVA) was used to compare continuous variables between dialysis patients and healthy subjects. The Mann-Whitney test was applied to analyze categorical data and to compare the difference in alterations of the D310 mononucleotide repeat between dialysis and healthy control subjects. On the basis of the least significant difference (LSD) method, ANOVA with a post hoc test was used to examine differences in TBARS and free thiol contents and human leukocyte mtDNA copy number among the 4 D310 repeat groups (7-C, 8-C, 9-10C, and T-to-C transition) in dialysis patients or control subjects. The level of significance was set to *P *< 0.05. Values were expressed as means ± standard deviation (SD).

## Results

No significant differences were observed in age and gender between dialysis patients and control subjects. Systolic blood pressure of dialysis patients was higher than in control subjects, but this difference only reached marginal statistical significance. Diastolic blood pressure of dialysis patients was lower than in control subjects. Comparison of biochemical data revealed lower levels of albumin, cholesterol, and sodium in dialysis patients as compared to control subjects. Levels of oxidative stress variable markers and levels of blood TBARS and mtDNA copy number were higher in dialysis patients than in control subjects, while blood free thiol levels were lower in dialysis patients than in control subjects (Table 1).

**Table 1:** Characteristics of demographic, biochemistry, and oxidative stress marker variables in dialysis and healthy subjects

	**Dialysis patients**	**Control group**	
		
	***N *= 184**	***N *= 213**	***P*-value**
Age	48.3 ± 14.2	49.2 ± 13.6	0.495
Gender (male)	71	83	0.938
BMI	22.1 ± 3.4	23.8 ± 3.7	0.000
Systolic BP (mmHg)	134 ± 25	130 ± 20	0.058
Diastolic BP (mmHg)	77 ± 14	82 ± 12	0.000
TBARS (μM/L)	1.126 ± 0.628	0.771 ± 0.478	0.000
Free thiol (μM/L)	1.515 ± 0.457	2.110 ± 0.423	0.000
mtDNA copy number	1664 ± 2551	412 ± 270	0.000
Hemoglobin (*g*/dl)	10.2 ± 1.7	14.0 ± 1.5	0.000
Hematocrit (%)	31.1 ± 5.4	42.2 ± 4.0	0.000
BUN (mg/dl)	68.6 ± 18.1	13.9 ± 4.2	0.000
Creatinine (mg/dl)	12.5 ± 2.7	1.0 ± 0.2	0.000
Sodium (mEq/L)	137 ± 4	141 ± 2	0.000
Potassium (mEq/L)	4.4 ± 0.8	4.2 ± 0.3	0.001
Calcium (mg/dl)	9.3 ± 1.0	9.1 ± 0.4	0.006
Phosphate (mg/dl)	5.1 ± 1.5	3.5 ± 0.5	0.000
Albumin (*g*/dl)	3.8 ± 0.4	4.3 ± 0.3	0.000
Blood sugar (mg/dl)	113 ± 47	101 ± 33	0.004
Total cholesterol (mg/dl)	190 ± 34	200 ± 37	0.006
Triglyceride (mg/dl)	162 ± 127	132 ± 133	0.018

Data presented as means ± standard deviation (SD) or (%). The Mann-Whitney test was used for ANOVA to compare groups (p < 0.05). TBARS, thiobarbituric acid reactive substances; mtDNA, mitochondrial DNA.

Comparison demographic, biochemistry, oxidative stress markers

variables in dialysis and healthy subjects. Lower levels of albumin, cholesterol, and sodium in the dialysis patients as compared to the control subjects. The levels of oxidative stress variables markers and levels of blood TBARS and mtDNA copy numbers were higher in the dialysis patients than in the control subjects, while the blood free thiol levels were lower in the dialysis patients than the control subjects

### Alterations in D310 mononucleotide repeat

**Figure 1:**
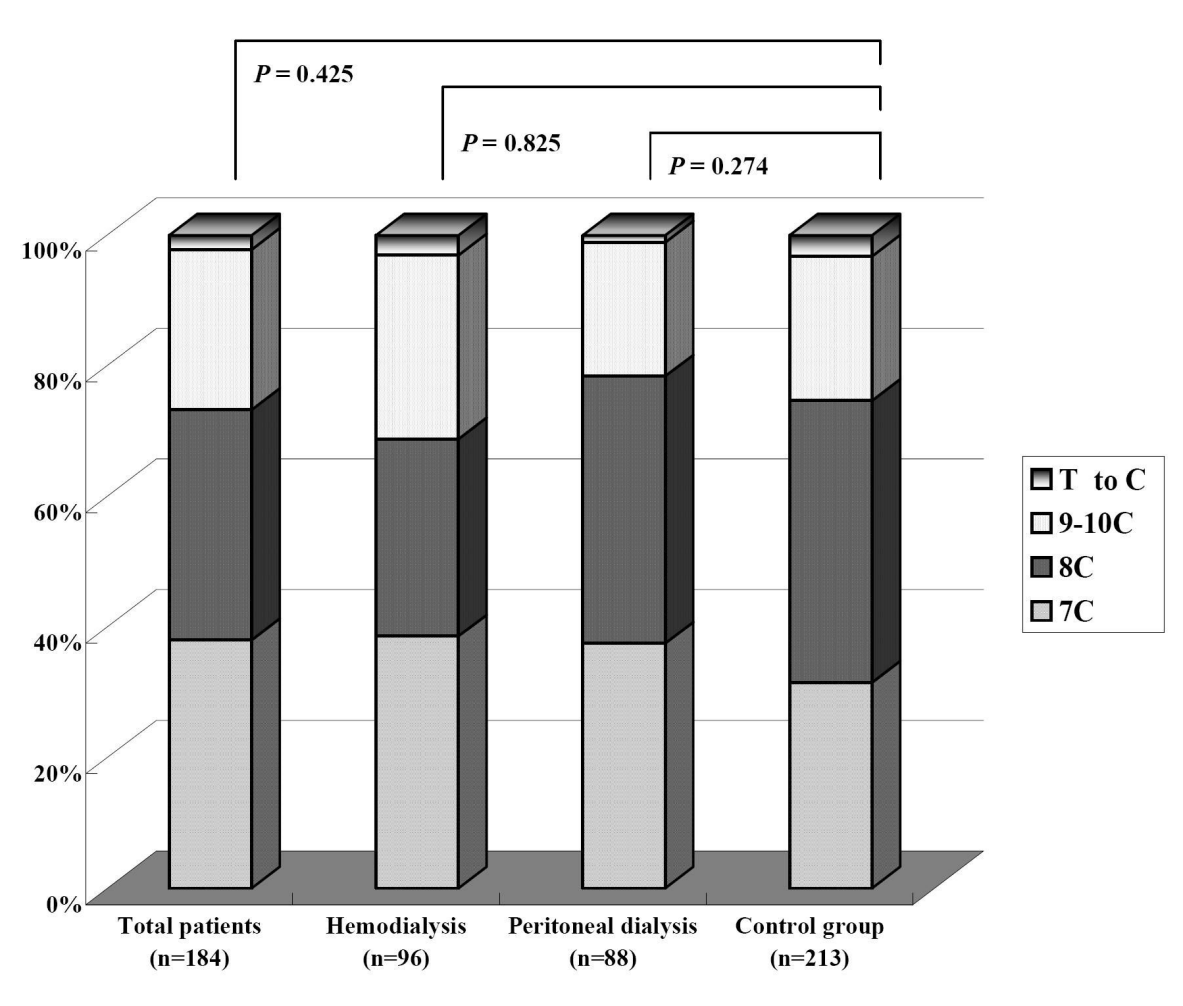
**Comparison of difference in alterations in D310 mononucleotide repeat between dialysis and healthy control subjects**. *Mann-Whitney test. T to C: T-to-C transition.

In D310 mononucleotide repeat sequencing analysis, variants can range at least from 7-C to 10-C among study subjects. 7-C and 8-C were common D310 repeat alterations in both dialysis patients and control subjects, and 8-C was the most common in the control cohort. In contrast, 7-C was the most common repeat alteration in the dialysis patients. The distribution of 7-C, 8-C, and 9-10C in the dialysis and control subjects was as follows: 7-C, 38% (*n *= 70) *vs. *31.5% (*n *= 67); 8-C, 35.3% (*n *= 65) *vs. *43.2% (*n *= 92); 9-10C, 24.5% (*n *= 45) *vs. *22.1% (*n *= 47). T-to-C transition was found in a few of the dialysis patients and control subjects, 2.2% (*n *= 4) *vs. *3.2% (*n *= 7) (Fig. 1).

### Association of D310 mononucleotide repeat with oxidative stress biomarkers and mitochondrial DNA copy number

The study subjects were categorized into 4 subgroups depending on D310 alterations, and significant differences were observed in the levels of oxidative stress biomarkers and mtDNA copy number between dialysis patients and control subjects in the 4 subgroups (Table 2). Analysis of variables by ANOVA, with the post hoc test based on the LSD method, revealed that a difference in D310 alterations (7-C to 10-C) did not result in a significant difference in levels of oxidative stress biomarkers and the mtDNA copy number in dialysis patients.

**Table 2:** Oxidative stress markers, and mtDNA copy number according to D310 mononucleotide repeat

	**D310 repeat groups**	**Dialysis patients (*N *= 184)**	**Control group (*N *= 213)**	***P*-value**
7-C		*n *= 70	*n *= 67	
	TBARS (μM/L)	1.110 ± 0.633	0.763 ± 0.423	0.000
	Free thiol (μM/L)	1.463 ± 0.460	2.185 ± 0.450	0.000
	mtDNA copy number	1638 ± 2263	423 ± 295	0.000

8-C		*n *= 65	*n *= 9	
	TBARS (μM/L)	1.205 ± 0.635	0.797 ± 0.530	0.000
	Free thiol (μM/L)	1.500 ± 0.461	2.064 ± 0.420	0.000
	mtDNA copy number	1783 ± 2602	416 ± 263	0.000

9-10C		*n *= 45	*n *= 47	
	TBARS (μM/L)	1.060 ± 0.625	0.752 ± 0.535	0.013
	Free thiol (μM/L)	1.607 ± 0.424	2.099 ± 0.396	0.000
	mtDNA copy number	1594 ± 3023	374 ± 257	0.007

T-to-C transition		*n *= 4	*n *= 7	
	TBARS (μM/L)	0.863 ± 0.454	0.634 ± 0.199	0.268
	Free thiol (μM/L)	1.630 ± 0.704	2.060 ± 0.356	0.203
	mtDNA copy number	933 ± 725	499 ± 215	0.161

Data (means ± SD) on post hoc analysis of TBARS, free thiol, and mtDNA copy number in same group, either dialysis or control groups. All were nonsignificant. TBARS, thiobarbituric acid reactive substances; mtDNA, mitochondrial DNA.

Correlation of D310 mononucleotide repeat and oxidative stress markers, mtDNA copy number. The study subjects were categorized into 4 subgroups depending on D310 alterations, and significant differences were observed in the levels of oxidative stress biomarkers and mtDNA copy number between the dialysis patients and the control subjects of the 4 subgroups. Analysis of the variables by ANOVA with the post hoc test based on the LSD method revealed that a difference in the D310 alterations (7-C to 10-C) did not result in significant difference in the levels of oxidative stress biomarkers and mtDNA copy number in the dialysis patients.

## Discussion

An increased oxidative burden is observed in chronic renal failure patients [1]. The onset of this condition is during the pre-dialysis period, and it is particularly remarkable after dialysis therapy is initiated [1,2]. Our previous investigations showed that CAPD patients have higher oxidative stress than healthy subjects, and this elevated oxidative stress leads to alterations in the mtDNA copy number in peripheral leukocytes [2]. In the present study, we demonstrated that dialysis patients, either on CAPD or HD, have increased blood levels of oxidative stress biomarkers and an increased mtDNA copy number in peripheral leukocytes. Since mitochondria are a major source of ROS and are highly susceptible to oxidative damage, we intend to further investigate the possible link between oxidative stress and mtDNA mutations.

mtDNA deletions/insertions and base changes mainly involve purine transitions, which have been assumed to occur after the action of ROS [11]. Many common polymorphisms in mtDNA accumulate in the D-loop [12]. The D-loop functions as a promoter for both heavy and light strands of mtDNA. A mononucleotide repeat between nucleotides 303 and 316-318 (D310) has recently been identified as a frequent hotspot for mutations in primary tumors [3]. Exposure to the oxidant *tert*-butyl hydroperoxide has been shown to induce mutations in the D310 region; this finding may explain the high frequency of homoplasmic D310 somatic mutations in many types of tumors [13]. The number of cytosines in the first stretch of the D310 region can vary from 7 to 9 in normal individuals. The homopolymeric C-stretch is part of CSBII, located within the regulatory D-loop region, and it is involved in the formation of a persistent RNA-DNA hybrid that leads to the initiation of mtDNA heavy-strand replication [14]. It is generally accepted that mtDNA mutations are generated during oxidative phosphorylation through pathways involving ROS. We therefore hypothesized that mtDNA mutations in the D-310 region would be frequently detected in chronic renal failure patients. Further, we expected somatic mutations in the D-310 region to be associated with a change in the mtDNA copy number of these patients.

In the present study, we found that 7-C and 8-C were common variants in D-310 repeat in both the dialysis patients and healthy subjects. This finding was identical to constitutive polymorphisms, described in a previous study on tumors [3]. Although the mtDNA copy number was higher in the dialysis patients than in the control subjects, a significant association between D-310 repeat alterations and mtDNA copy number was not observed in dialysis patients. Previous investigations have well established that somatic mtDNA mutations accumulate with age; however, the levels of any single mtDNA mutation are too low to cause significant tissue pathology [15]. We proposed that chronic renal failure was not the result of just 1 type of mtDNA damage but rather the sum total of a large number of mtDNA mutations that accumulate concurrently over time. This assumption may explain our findings on the association between the mtDNA copy number and D-310 repeat alterations.

## Conclusion

In conclusion, dialysis patients had more oxidative stress than healthy subjects. Although oxidative stress led to a compensatory increase in the mtDNA copy number in dialysis patients, alterations of D-310 repeat were not associated with the mtDNA copy number. Finally, D-310 repeat alterations were not significantly different between dialysis patients and healthy subjects. On the basis of our findings, we have proposed a possible gateway to further investigate the association between mtDNA mutations and oxidative stress in chronic renal failure.

## Competing interests

The authors declare that they have no competing interests.

## Authors' contributions

J-BC participated in the design of the study and drafted the manuscript. T-KL provided the intellectual content of critical importance to the investigation and laboratory assessment. W-CL performed measurement of mtDNA copy number. L-CL collected clinical data of subjects. C-WL performed measurement of oxidative stress biomarkers. P-WW assisted in the design of the study. M-MT assisted in the drafting of the manuscript. S-CL performed statistical analysis.
